# Evaluation of intraoperative color Doppler ultrasonography in the surgical evacuation of acute intracranial hematoma: a single-center study from Botswana

**DOI:** 10.3389/fsurg.2026.1764756

**Published:** 2026-05-20

**Authors:** Yang Zhang, Jianxiang Zhao, Bayela Nfila, Dorcas Kambai Mufalali, Shaoya Yin

**Affiliations:** 1 Clinical College of Neurology, Neurosurgery and Neurorehabilitation, Tianjin Medical University, Tianjin, China; 2 Department of Neurosurgery, Fuzhou University Affiliated Provincial Hospital, Fuzhou, Fujian, China; 3 Department of Critical Care Medicine, Fuzhou University Affiliated Provincial Hospital, Fuzhou, Fujian, China; 4 Department of Neurosurgery, Nyangabgwe Referral Hospital, Francistown, Botswana; 5 Department of Neurosurgery, Huanhu Hospital, Tianjin, China

**Keywords:** acute intracranial hemorrhage, Botswana, color Doppler ultrasonography, hematoma clearance, intraoperative imaging

## Abstract

**Background:**

Achieving accurate intraoperative localization of intracranial hematomas is essential for maximizing evacuation efficiency and minimizing neurological injury. Conventional preoperative CT provides static imaging and cannot reflect dynamic hematoma evolution during surgery, particularly in acute settings.

**Objective:**

To evaluate the effectiveness of intraoperative color Doppler ultrasonography in improving hematoma clearance and neurological outcomes in patients undergoing craniotomy for acute intracranial hemorrhage, and to assess its feasibility in resource-limited environments.

**Methods:**

In this prospective randomized study, 57 patients with acute supratentorial intracranial hematomas (including epidural, subdural, and intraparenchymal hemorrhages) were allocated to either an observation group receiving intraoperative color Doppler ultrasonography guidance (*n* = 34) or a control group undergoing conventional surgery based on preoperative CT alone (*n* = 23). Hematoma clearance rate, Glasgow Coma Scale (GCS), National Institutes of Health Stroke Scale (NIHSS), and length of hospital stay were compared between groups.

**Results:**

The proportion of patients achieving >90% hematoma clearance was significantly higher in the ultrasound-guided group (70.6% vs. 26.1%, *P* < 0.001). Two weeks postoperatively, the observation group demonstrated better neurological recovery, with higher GCS scores [12 (10–12) vs. 9 (8–10), *P* < 0.001] and lower NIHSS scores (8.38 ± 2.15 vs. 11.78 ± 2.15, *P* < 0.001). The ultrasound-guided group also had a shorter hospital stay (12.47 ± 5.34 vs. 16.39 ± 7.38 days, *P* = 0.024). Intraoperative ultrasonography successfully detected residual, newly formed, and contralateral hematomas that were not identified on preoperative CT.

**Conclusion:**

Intraoperative color Doppler ultrasonography enables precise, real-time hematoma localization. It provides additional dynamic information beyond static preoperative imaging, potentially improving surgical clearance rates and short-term neurological outcomes in patients with acute supratentorial hematomas. This technique is a practical adjunct to CT-guided neurosurgery, particularly in resource-constrained settings.

## Introduction

Acute intracranial hemorrhage is a common and life-threatening condition in neurosurgery, characterized by sudden onset, severe clinical manifestations, high mortality, and poor prognosis (1). Intracranial hemorrhage can be categorized into traumatic and spontaneous intracranial hemorrhage. Regardless of the cause, hematoma evacuation should be performed promptly once the surgical indications for craniotomy are met. The goal of surgery is to relieve compression of surrounding brain tissue and critical functional areas, reduce intracranial pressure (ICP), and mitigate the space-occupying effect, thus preventing neurological deterioration. Without timely intervention to reverse signs of transtentorial herniation, patients are likely to progress to central herniation, which is almost universally fatal (2). Accurate positioning of the hematoma before and during the operation plays a vital role in the effect of the operation and the prognosis of the patients no matter which kind of surgical methods are adopted (3, 4). Brain CT can objectively and accurately locate the position, shape, and volume of intracranial hematoma. However, the size and immobility of CT equipment limit its intraoperative use, making it impractical to obtain updated scans during surgery (5). Because acute intracranial hemorrhage progresses rapidly, intraoperative findings often differ from the preoperative CT. Surgeons may encounter newly formed hematomas not visible on initial imaging or unexplained brain swelling, both of which may indicate delayed hematoma formation. Under such conditions, repositioning, and dynamic monitoring of the hematoma during surgery are critically important. Color Doppler ultrasonography is a practical choice as it allows continuous monitoring of hematoma changes, offers unmatched portability and real-time feedback, and can be used intraoperatively (6, 7).

This study aims to evaluate the clinical utility of intraoperative color Doppler ultrasonography in guiding the surgical clearance of acute intracranial hematoma. Furthermore, given that our hospital—located in north-central Botswana—serves as a major neurosurgical referral center managing nearly half of such cases nationwide, we also explore the feasibility and potential advantages of applying this technique within resource-limited healthcare settings.

## Materials and methods

### Data collection

This was a prospective, randomized controlled study conducted at our hospital, Francistown, Botswana, between January 2021 and January 2023. Ethical approval was obtained from the hospital's Research and Ethics Committee (Project ID: 202123), and written informed consent was obtained from all participants or their legal guardians.

A total of 57 patients with acute supratentorial intracranial hematomas (epidural, subdural, or intraparenchymal), confirmed by CT, were consecutively recruited during the study period. To minimize confounding factors, inclusion criteria were as follows: age between 18 and 60 years (mean age: 42.6 years); no history of diabetes mellitus or severe cardiac, pulmonary, hepatic, or renal dysfunction; and no structural vascular abnormalities such as aneurysms or arteriovenous malformations (excluded based on preoperative or postoperative imaging findings). Patients with isolated intraventricular hemorrhage or posterior fossa hemorrhage were excluded due to the limited accessibility and imaging constraints of intraoperative ultrasonography in these regions. All patients underwent cranial CT prior to surgery for hematoma localization and surgical planning.

Randomization and allocation. Participants were randomized using computer-generated permuted blocks with a 1:1 target allocation. Allocation was concealed with sequentially numbered, opaque, sealed envelopes prepared by an independent coordinator; envelopes were opened after eligibility confirmation in the operating room. Over-enrolment was planned to offset perioperative attrition and to maintain the minimum per-group sample size determined by the power analysis. The observation group (*n* = 34; 22 males, 12 females; mean age: 43.2 years) underwent craniotomy assisted by intraoperative color Doppler ultrasonography in addition to preoperative CT. The control group (*n* = 23; 14 males, 9 females; mean age: 40.2 years) received conventional surgery based only on preoperative CT. On admission, Glasgow Coma Scale (GCS) scores were distributed as follows: in the observation group, 13 patients scored 3–5, 16 scored 6–8, and 5 scored 9–12; in the control group, 9 scored 3–5, 9 scored 6–8, and 5 scored 9–12.

The required sample size was calculated using G*Power version 3.1 (Heinrich Heine University Düsseldorf, Germany). The power analysis (two-sided *α*=0.05, power=0.80) indicated ≥23 per group based on the expected difference in the proportion achieving >90% clearance. To accommodate potential attrition and exclusions after allocation, we over-enrolled participants, anticipating that not all patients would meet intraoperative eligibility. Ultimately, 34 patients remained in the observation group and 23 in the control group, both meeting the calculated minimum sample size.

### Procedure

#### Preparation for operation

All patients underwent general anesthesia. The SonoSite X-PORTE color Doppler ultrasound diagnostic system (Fujifilm, USA; portable model) was used for intraoperative imaging in the observation group. A linear array intraoperative ultrasound probe (the width is approximately 25–38 millimeters.) was used. The frequency range was 5–8 MHz. The size of this probe matched that of a standard craniotomy window (≥3 centimeters), enabling it to be directly placed on the cortical bone surface for real-time imaging. After applying a sterile saline coupling agent, the probe was wrapped with a sterile film to ensure no bubbles remained. The probe was applied directly to the brain surface after dura mater incision. A bone window of at least 3 cm in diameter was created to accommodate the ultrasound probe. Preoperative brain CT was performed in all patients and used to determine the surgical plan, including the craniotomy site and trajectory to the hematoma.

All intraoperative ultrasonography procedures were performed by neurosurgeons who had received prior training in intraoperative ultrasound techniques. Specifically, the operators had at least 3–5 years of experience in neurosurgical practice and had undergone focused training in intraoperative ultrasonography, including both theoretical instruction and supervised hands-on practice.

Before the initiation of this study, operators completed a short training period involving approximately 10–15 supervised cases to become familiar with probe handling, image acquisition, and interpretation of ultrasound findings in intracranial hematomas. The learning curve for basic intraoperative ultrasound application was relatively short, and surgeons were generally able to achieve reliable image acquisition and interpretation after this initial training phase. To minimize inter-operator variability, all scans were performed or supervised by at least one experienced operator, and imaging findings were interpreted in conjunction with intraoperative observations.

#### Surgical procedures

All patients underwent craniotomy for hematoma evacuation. In the observation group, after standard sterile preparation and draping, the bone window was opened based on hematoma localization observed on preoperative CT. Following initial removal of epidural or subdural hematomas and visible clots, intraoperative ultrasonography was performed using both B-mode and color Doppler modes to assess the presence, depth, and location of any remaining hematoma. For color Doppler imaging, the pulse repetition frequency (PRF) was set at 1.5–3.0 kHz, the wall filter was maintained at a low level (50–100 Hz) to detect low-velocity intracranial blood flow, and the color gain was adjusted just below the noise threshold to optimize signal sensitivity while avoiding artifacts. The color box size and angle were manually adjusted to focus on regions of interest, particularly around the hematoma margins and major cerebral vessels. The scanning depth is approximately 6–8 centimeters, which is sufficient for the ultrasound waves to penetrate fully, allowing for clear observation of the structures of the ipsilateral and contralateral cerebral hemispheres through the craniotomy window. By adjusting the direction of the probe (tilting and rotating in the axial and coronal planes), the ultrasound beam can pass through the midline, enabling the identification of the hematoma on the opposite side. The patient's head position (supine or lateral position) is adjusted according to the location of the hematoma to optimize the approach. The contralateral lesion is identified as a high-echo or mixed-echo area outside the midline, usually associated with displacement of the midline structures and asymmetry of the ventricles. The midline structures, including the third ventricle and falx cerebri, were used as anatomical landmarks to guide orientation and confirm contralateral localization.

Ultrasonography was also employed to identify intraoperative brain bulging or newly formed hematomas, enabling the surgeon to adjust the evacuation route in real time. After hematoma evacuation, a final ultrasound scan was conducted to confirm the absence of residual hematoma or active bleeding.

In the control group, conventional craniotomy and hematoma evacuation were performed based on preoperative CT imaging and anatomical landmarks, without the use of intraoperative ultrasonography.

To evaluate the reliability of intraoperative ultrasonography, qualitative comparisons were performed between preoperative CT findings and intraoperative ultrasound images, as well as between intraoperative ultrasound and postoperative CT scans. Key parameters assessed included hematoma location, size approximation, presence of residual hematoma, and detection of newly formed or contralateral lesions. Due to the lack of volumetric software, these comparisons were primarily descriptive rather than quantitative.

#### Hematoma classification and location

Hematomas were classified according to both type and anatomical location based on preoperative CT imaging. The included types comprised epidural hematoma, subdural hematoma, and intraparenchymal hemorrhage. Anatomically, hematomas were categorized as lobar (frontal, temporal, parietal, occipital) or deep (basal ganglia region). The applicability of intraoperative ultrasonography was primarily limited to supratentorial hematomas accessible through the craniotomy window. Deep-seated hematomas required careful probe angulation but remained detectable due to sufficient ultrasound penetration depth (6–8 cm). In contrast, lesions located in the posterior fossa or isolated intraventricular regions were not included due to acoustic window limitations. The effectiveness of intraoperative ultrasonography may vary depending on hematoma location, with superficial and lobar hematomas being more readily visualized, while deep hematomas required more operator experience for accurate localization.

#### Postoperative treatment

After surgery, all patients were admitted to the neurosurgical intensive care unit for close monitoring. Brain CT scans were routinely performed 24–72 h postoperatively to evaluate hematoma clearance and detect any early complications. Neurological status was assessed regularly, including consciousness, pupillary response, and observation for swelling at the surgical site or signs of papilledema. Supportive care included sedation, dehydration therapy, and other standard neurosurgical treatments to manage ICP and promote recovery.

#### Outcome measures

Four key clinical outcome indicators were assessed in both groups: (1) Hematoma clearance rate: Postoperative CT scans were reviewed by two independent neurosurgeons blinded to group allocation. The clearance rate was calculated as: (original hematoma volume—residual hematoma volume)/ original hematoma volume. To facilitate comparison, clearance was categorized as >90%, 60%–90%, or <60%, based on visual CT estimation and clinical documentation. (2) Level of consciousness: The GCS was used to assess patients' eye-opening, verbal, and motor responses, each scored 4, 5, and 6 points respectively (total score range: 3–15). A score of 15 indicated full consciousness; 12–14 indicated lethargy; 8–11 indicated mild coma; 4–8 indicated coma; 3 indicated deep coma. GCS was recorded preoperatively, and again on postoperative day 3 and day 14. (3) Neurological function: Neurological deficits was assessed using the National Institutes of Health Stroke Scale (NIHSS), which assesses 11 domains with a total score ranging from 0 (normal) to 42 (severe deficits). Assessments were conducted within 24 h before surgery and again at two weeks postoperatively. (4) Length of hospital stay: Total length of stay (in days) from admission to discharge was recorded for each patient.

#### Statistical analysis

All statistical analyses were performed using SPSS 25.0 software (IBM, USA). Continuous variables were tested for normality using the Shapiro–Wilk test. Normally distributed data were expressed as mean and standard deviation (SD) and compared between groups using the independent-samples *t*-test. Non-normally distributed data were presented as median and interquartile range (IQR) and compared using the Mann–Whitney U test. Categorical variables were expressed as counts (n) and percentages (%) and compared using the chi-square test or Fisher's exact test where appropriate. *P* < 0.05 indicated that the difference was statistically significant.

## Results

### Enhanced hematoma clearance and recovery with ultrasound-guided surgery

As shown in Table 1, the two groups were comparable at baseline in terms of age, sex distribution GCS scores, and NIHSS scores upon admission (*P* > 0.05), indicating balanced preoperative characteristics.

**Table 1:** Baseline characteristics and postoperative outcomes between observation and control groups.

Groups	Observation group (*n* = 34)	Control group (*n* = 23)	t//z/χ^2^	*P*-value
Age (years)	43.29 ± 8.54	40.26 ± 8.09	1.34	0.185
Gender			0.09	0.768
Male	22 (65%)	14 (61%)		
Female	12 (35%)	9 (39%)		
Length of stay (Day)	12.47 ± 5.34	16.39 ± 7.38	−2.33	0.024
GCS on admission			0.59	0.746
3–5 points	13	9		
5–8 points	16	9		
9–12 points	5	5		
GCS (score)				
Before operation (IQR)	5 (4,6)	5 (5,6)	−0.24	0.808
2 weeks after operation (IQR)	12 (10,12)	9 (8, 10)	−4.88	<0.001
NIHSS (score)				
Before operation	27.53 ± 3.40	27.43 ± 3.58	0.10	0.920
2 weeks after operation	8.38 ± 2.15	11.78 ± 2.15	<0.001	−5.86
Clearance rate			−3.638	<0.001
>90%	24 (70.6)	6 (26.1)		
60%—90%	7 (20.1)	6 (26.1)		
<60%	3 (9.3)	11 (47.9)		

GCS score, Glasgow Coma Scale (GCS) scores; NIHSS score, National Institutes of Health Stroke Scale.

Two weeks after surgery, however, the observation group showed significantly better neurological recovery and shorter hospitalization time. The GCS score was higher in the observation group [median 12 (IQR: 10–12)] compared to the control group [median 9 (IQR: 8–10)] (*P* < 0.001). Similarly, the NIHSS score was significantly lower in the observation group (8.38 ± 2.15) than in the control group (11.78 ± 2.15) (*P* < 0.001). In addition, the average length of hospital stay was significantly shorter in the observation group (12.47 ± 5.34 days) than in the control group (16.39 ± 7.38 days) (*P* = 0.024).

With regard to hematoma clearance, a significantly greater proportion of patients in the observation group achieved a clearance rate >90% (70.6%) compared to the control group (26.1%) (*P* < 0.001).

These findings suggested that intraoperative application of color Doppler ultrasonography might enhance hematoma removal efficiency, improved neurological outcomes, and reduced hospitalization time.

In general, intraoperative ultrasonography findings were qualitatively consistent with preoperative CT in terms of hematoma location and extent. In most cases, ultrasound accurately identified the primary hematoma location and provided additional real-time information such as residual or newly formed hematomas that were not evident on preoperative CT. Moreover, postoperative CT confirmed that intraoperative ultrasound was effective in identifying residual hematoma and newly developed or contralateral lesions, as illustrated in the representative cases.

#### Representative case 1

**Figure 1:**
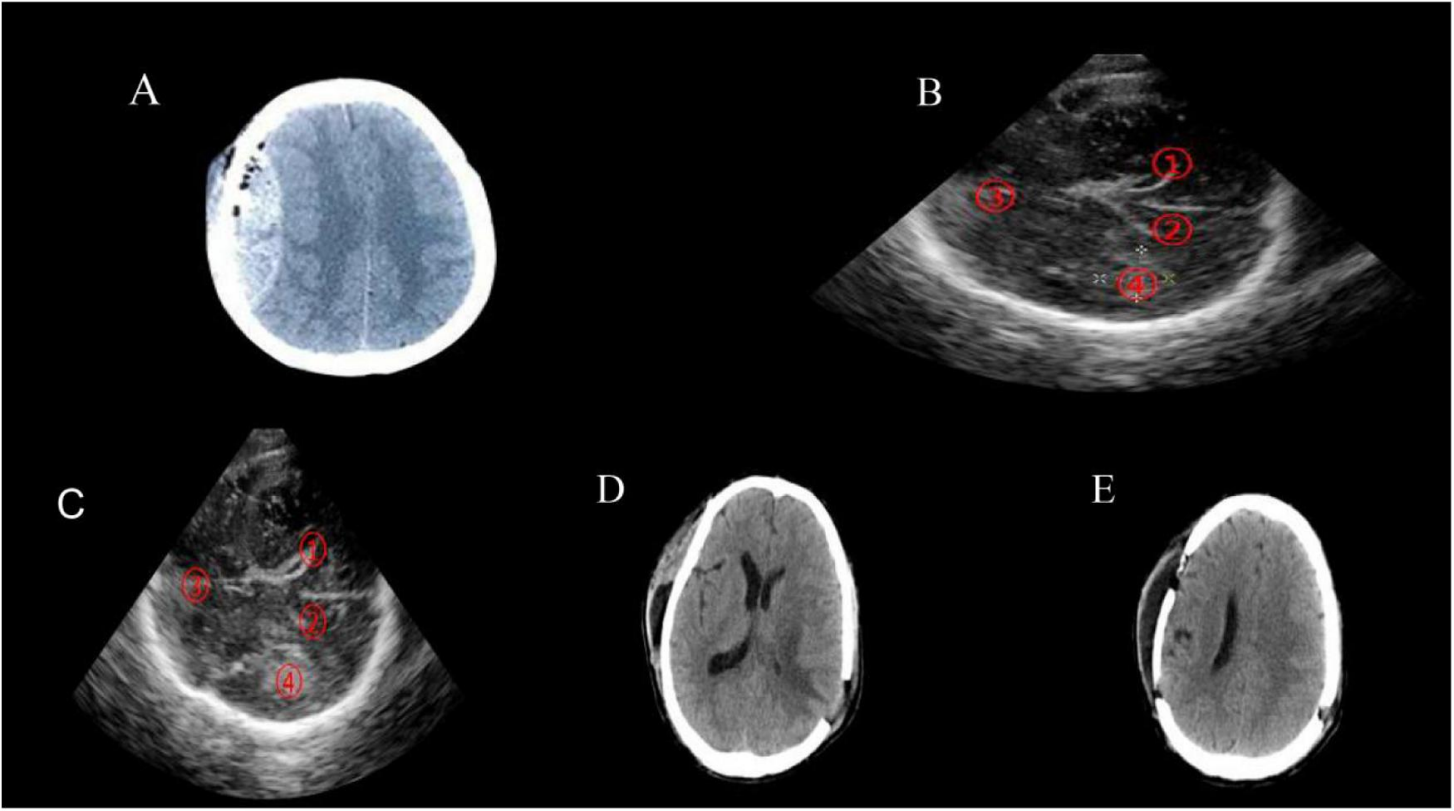
Brain CT image of case 1. **(A,B)** The patient was admitted to our hospital 1 h after the acute head injury. Brain CT showed a right temporoparietal epidural hematoma. **(C,D)** Ultrasonography of the brain was performed intra-operatively and the results showed a left temporal intracerebral hematoma [(1) the right posterior horn of the lateral ventricle. (2) the left posterior horn of the lateral ventricle. (3) the midline structure. (4) the hyper-echo area of the left temporal lobe might indicate the intracerebral hematoma of the left temporal lobe]. **(E,F)** After the skull was immediately closed and the family members of the patients were informed, the intracerebral hematoma was removed by small bone window craniotomy in the left temporal region. The pupil was contracted after surgery.

A patient was admitted one hour after acute head injury. CT revealed a right temporoparietal epidural hematoma with unequal pupils and absent light reflex, suggesting brain herniation (Figures 1A,B). Emergency craniotomy was performed, and although the hematoma was cleared, unexplained brain swelling was observed. Intraoperative color Doppler ultrasonography revealed a contralateral left temporal intracerebral hematoma (Figures 1C,D). A second craniotomy via a small bone window was immediately conducted. Postoperative CT on day 9 confirmed complete removal of bilateral hematomas (Figures 1E,F), illustrating the critical role of intraoperative ultrasound in detecting delayed or contralateral lesions.

#### Representative case 2

**Figure 2:**
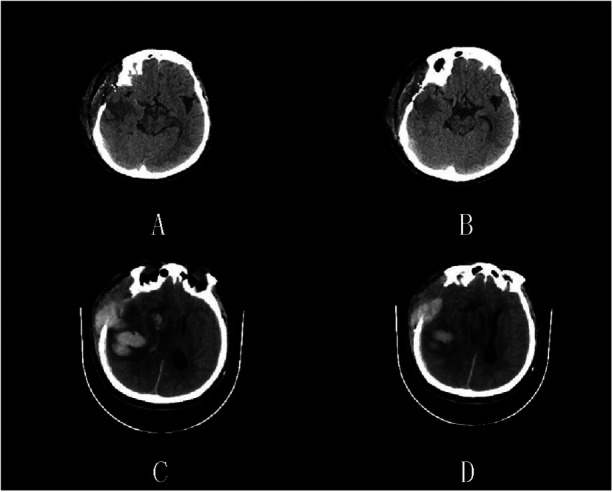
On the 7th postoperative day of case 2, the brain CT images were re examined. **(A,B)** Brain CT examination on the 7th day after surgery showed complete removal of intracerebral hematoma in the right temporal lobe. **(C,D)** The patient's brain CT emergency examination showed an intracerebral hematoma in the right temporal lobe.

**Figure 3:**
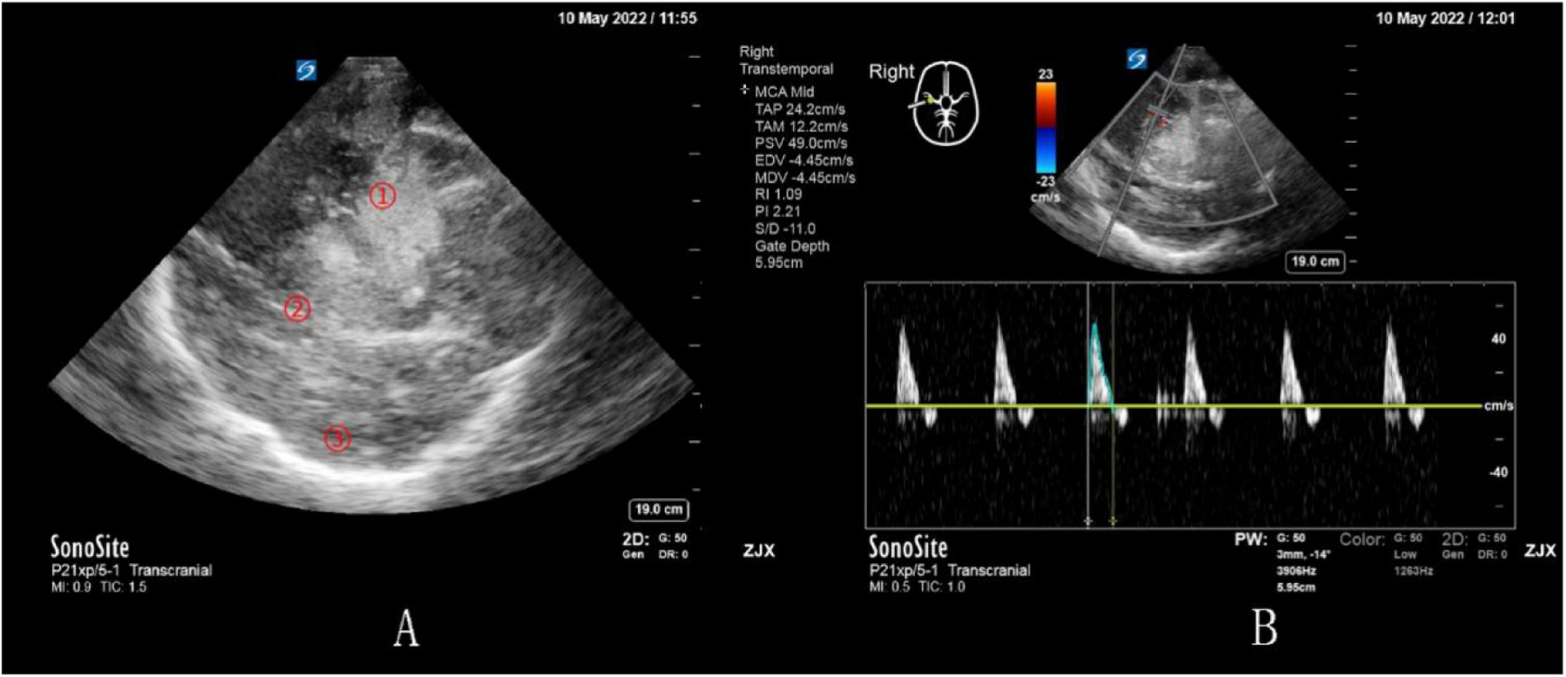
The right brain image of the patient was examined by bedside color Doppler ultrasound on day 9 after surgery. **(A)** The patient's midline structure shifted to the left and the contralateral periosteum showed a hyperechoic region, suggesting increased intracranial pressure on the left side [(1) hyperechoic hematoma in ultrasonography. (2) hyperechoic midline structure, visible midline shift. (3) hyperechoic contralateral periosteum]. **(B)** Color Doppler ultrasonography of the right middle cerebral artery reveals reversed diastolic flow and reduced peak systolic velocity (49 cm/s), suggesting compromised cerebral perfusion and elevated intracranial pressure. Pulsatility index (PI) was 2.21, indicating abnormally high vascular resistance.

A patient underwent surgery for spontaneous hemorrhage in the right external capsule area and initially recovered well (GCS 14; E4V5M5). On postoperative day 7, brain CT confirmed complete clearance of the hematoma (Figures 2A,B), but the pulsatility index was elevated (PI = 2.21), indicating cerebral hypoperfusion. On day 9, the patient developed neurological deterioration, with decreased GCS and sluggish pupillary light reflex. Emergency CT revealed a recurrent right temporal intracerebral hematoma (Figures 2C,D). Bedside Doppler ultrasonography showed a new hyperechoic hematoma, midline shift, and hyper-echogenic contralateral periosteum (Figure 3A). Furthermore, reversed diastolic flow and reduced systolic velocity (49 cm/s) were observed in the middle cerebral artery (Figure 3B), indicating critical hemodynamic compromise.

## Discussion

Accurate localization of hematoma is essential not only for maximizing hematoma evacuation but also minimizing collateral damage to surrounding normal brain tissue (8). To date, the main surgical techniques for hematoma clearance include microsurgical evacuation, neuroendoscopy-assisted evacuation, stereotaxic aspiration, and trepanation with drainage (8). Among them, neuroendoscopic evacuation is associated with a high clearance rate, faster recovery, and improved long-term prognosis (9). Regardless of the method used, successful hematoma removal requires precise intraoperative localization and continuous assessment, especially to prevent delayed hematoma formation or rebleeding (10).

Preoperative cerebral CT remains the gold standard for hematoma visualization, allowing evaluation of location, shape, and surrounding edema (11). However, in acute settings, the dynamic nature of intracranial hemorrhage presents a major challenge. As noted in recent studies, hematoma expansion occurs predominantly within the first 3–6 h after symptom onset, with the highest risk in the ultra-early period (12). This creates a mismatch between preoperative imaging and intraoperative findings. Surgeons may encounter newly formed hematomas or unexpected brain swelling not visible on preoperative CT. These discrepancies highlight the need for real-time imaging to update surgical planning. This challenge becomes even more significant in cases involving deep brain regions such as the basal ganglia or thalamus, where repeated blind attempts at evacuation could result in severe neurological injury due to the density of critical structures (13). According to the literature, patients presenting with acute intracranial hemorrhage and unilateral pupillary dilation can often survive if surgical decompression is performed within one hour of onset (14). In contrast, delaying surgery beyond two hours after coma onset drastically reduces the chance of recovery (15). These findings support the rationale for integrating dynamic intraoperative imaging into emergency neurosurgical procedures. Importantly, the present study adds to the existing literature by providing prospective clinical evidence demonstrating the feasibility and practical utility of intraoperative color Doppler ultrasonography in real-world surgical settings. Unlike previous studies that focused primarily on technical feasibility or small case series, our study highlights its potential role in improving intraoperative decision-making and surgical efficiency, particularly in resource-limited environments where advanced intraoperative imaging modalities are not readily available.

In Botswana, there are only two public hospitals equipped with CT scanners, separated by approximately 500 kilometers. Emergency patient transfers often delay intervention due to machine downtime or transportation limitations (16). In such contexts, the introduction of intraoperative color Doppler ultrasonography provides a significant clinical advantage. It offers portable, real-time, and non-invasive assessment of intracranial structures and hemodynamics, enabling detection of residual hematomas, brain swelling, or newly developed lesions (17). Our findings are consistent with these advantages, particularly in cases where intraoperative ultrasound detected fresh or contralateral hematomas not visible in the initial CT scan, guiding timely surgical decisions and potentially avoiding secondary procedures.

The reliability of intraoperative ultrasound as a real-time imaging modality is further supported by its correlation with CT findings. In our study, ultrasound demonstrated good qualitative agreement with preoperative CT in hematoma localization and with postoperative CT in assessing residual clots. Although our study was limited to qualitative comparison, these observations are consistent with previous comparative studies, which have shown high correlations between ultrasound and CT in measuring key parameters such as midline shift (correlation coefficient *β* = 0.91), hematoma volume (*β* = 1.01), and ventricular width (*β* = 0.98) (18). Other studies have also reported good concordance between intraoperative ultrasound and preoperative MRI in tumor volume estimation (R = 0.99) and residual tumor detection (sensitivity 100%, specificity 84.6%) (19). The ability of ultrasound to detect dynamic changes not visible on static preoperative imaging—such as contralateral hematoma formation or intraoperative brain swelling—represents a distinct advantage (20). This real-time feedback allows surgeons to adapt their strategy promptly, potentially improving outcomes and reducing the need for secondary procedures. In resource-limited settings where advanced intraoperative imaging modalities such as CT or MRI are not readily available, intraoperative ultrasound provides a cost-effective and real-time alternative for surgical guidance (21, 22).

This technique has demonstrated its utility especially in deep-seated hematomas. Prior research has shown that Doppler-guided approaches reduce neurological complications by helping define the safest evacuation paths, minimizing injury to adjacent tissues (23). Additionally, intraoperative brain shifts caused by cerebrospinal fluid (CSF) release or craniectomy can alter hematoma positioning relative to preoperative imaging. Real-time ultrasound enables precise reassessment under these evolving conditions, consistent with prior reports (24).

An important consideration in the application of intraoperative ultrasonography is the potential influence of hematoma location on its effectiveness. In theory, deep-seated hematomas (e.g., basal ganglia or thalamic hemorrhages) may derive greater benefit from real-time ultrasound guidance, as blind attempts at evacuation in these eloquent regions carry a higher risk of neurological injury (13). Previous studies have demonstrated that intraoperative ultrasound-assisted neuroendoscopy can improve the efficacy and safety of treatment for hypertensive intracerebral hemorrhage in the basal ganglia region (25). Similarly, intraoperative ultrasound has been shown to be a reliable imaging tool for evacuation of basal ganglia/thalamic hematomas and intracerebral contusions (26). Conversely, superficial lobar hematomas might be adequately managed with preoperative CT alone, given their easier accessibility and lower risk of collateral damage during blind exploration. In our study, the observation group included a mix of superficial and deep hematomas, and ultrasound demonstrated utility across both categories. However, due to the limited sample size and the fact that detailed location-specific data were not pre-specified in the initial data collection protocol, we were unable to perform subgroup analyses to quantitatively assess whether the benefit of ultrasound varied by hematoma location. This represents a limitation of our study and should be addressed in future prospective investigations with larger cohorts and more rigorous data collection frameworks. Despite this limitation, our clinical experience suggests that intraoperative ultrasound is particularly valuable in deep-seated hematomas, where it helps define the safest evacuation trajectory and confirms complete removal without excessive tissue manipulation. For superficial hematomas, ultrasound remains useful for detecting residual clots or unexpected contralateral lesions, as illustrated in Case 1. Further studies are warranted to establish location-specific guidelines for the use of intraoperative ultrasound in intracranial hematoma evacuation.

Furthermore, in extreme scenarios where CT is unavailable—such as rural facilities or during equipment malfunction—ultrasound guidance, in conjunction with clinical neurological signs, can facilitate life-saving decompressive craniectomy, even without radiologic imaging support (27, 28). Such flexibility is of critical importance in low-resource healthcare environments. Our experience supports these findings, demonstrating that ultrasound can provide reliable, timely guidance when rapid intervention is required.

However, this study still has several limitations. Firstly, the current intraoperative ultrasound system lacks volume reconstruction capabilities, making it difficult to accurately measure the hematoma volume. Similarly, due to limited access to specialized software tools in the research environment, CT assessment of postoperative hematoma clearance relies on visual estimation rather than automated volume analysis. These limitations force us to adopt a grading and classification system (>90%, 60%–90%, <60%), which may reduce measurement accuracy. Additionally, patient prognosis may be influenced by various unmeasured confounding factors, such as preoperative hematoma volume, hematoma type and location, surgical technique, intraoperative bleeding, and postoperative complications. Due to the limited sample size, multivariate correction analysis and detailed subgroup analysis were not conducted, which limits our ability to assess potential imbalances in hematoma types between different groups. Thirdly, operator dependency is an inherent limitation (24). Although all operators in this study have 3–5 years of neurosurgical experience and have completed 10–15 cases of intensive training under the guidance of mentors, there may still be differences in image acquisition and interpretation. Fourthly, theoretically, excessive pressure applied by the probe on the cortical surface may cause additional mechanical damage, especially in fragile brain tissue. Maintaining strict sterility when operating the probe during surgery is crucial to avoid infection risks. Finally, ultrasound images may be affected by artifacts, especially in cases of cerebral edema, uneven hematoma, or air bubbles, all of which can lead to misjudgment. These factors should be carefully considered when applying intraoperative ultrasound examination in clinical practice. Future research should expand the sample size, conduct multicenter studies, and adopt stricter operational standards and data collection methods to further evaluate the role of intraoperative ultrasound color Doppler examination.

## Conclusion

In summary, intraoperative color Doppler ultrasonography appears to be a useful adjunct to CT-guided neurosurgery for acute intracranial hematomas. Its real-time adaptability and portability assist in surgical planning, intraoperative localization, and safety management, particularly in resource-constrained settings. However, given the potential influence of confounding factors and the modest sample size, these results should be interpreted with caution. Further large-scale, controlled studies with standardized protocols are warranted to confirm its clinical value and establish location-specific guidelines.

## Data Availability

The original contributions presented in the study are included in the article/Supplementary Material, further inquiries can be directed to the corresponding author.
